# A rapid review of the rate of attrition from the health workforce

**DOI:** 10.1186/s12960-017-0195-2

**Published:** 2017-03-01

**Authors:** Sofia Castro Lopes, Maria Guerra-Arias, James Buchan, Francisco Pozo-Martin, Andrea Nove

**Affiliations:** 1 ICS Integrare, calle Balmes 30,3-1a, 08007 Barcelona, Spain; 2 Queen Margaret University, Edinburgh, Scotland United Kingdom; 3 University of Technology, Sydney, Australia; 4 Department of Global Health and Development, Faculty of Public Health and Policy, London School of Hygiene and Tropical Medicine, 15-17 Tavistock Place, London, WC1H 1SH United Kingdom

**Keywords:** Human resources for health, Attrition, Brain drain, Losses, Health workforce, Equity, Universal health coverage, Turnover, Wastage, Retention

## Abstract

**Background:**

Attrition or losses from the health workforce exacerbate critical shortages of health workers and can be a barrier to countries reaching their universal health coverage and equity goals. Despite the importance of accurate estimates of the attrition rate (and in particular the voluntary attrition rate) to conduct effective workforce planning, there is a dearth of an agreed definition, information and studies on this topic.

**Methods:**

We conducted a rapid review of studies published since 2005 on attrition rates of health workers from the workforce in different regions and settings; 1782 studies were identified, of which 51 were included in the study. In addition, we analysed data from the State of the World’s Midwifery (SoWMy) 2014 survey and associated regional survey for the Arab states on the annual voluntary attrition rate for sexual, reproductive, maternal and newborn health workers (mainly midwives, doctors and nurses) in the 79 participating countries.

**Results:**

There is a diversity of definitions of attrition and barely any studies distinguish between total and voluntary attrition (i.e. choosing to leave the workforce). Attrition rate estimates were provided for different periods of time, ranging from 3 months to 12 years, using different calculations and data collection systems. Overall, the total annual attrition rate varied between 3 and 44% while the voluntary annual attrition rate varied between 0.3 to 28%. In the SoWMy analysis, 49 countries provided some data on voluntary attrition rates of their SRMNH cadres. The average annual voluntary attrition rate was 6.8% across all cadres.

**Conclusion:**

Attrition, and particularly voluntary attrition, is under-recorded and understudied. The lack of internationally comparable definitions and guidelines for measuring attrition from the health workforce makes it very difficult for countries to identify the main causes of attrition and to develop and test strategies for reducing it. Standardized definitions and methods of measuring attrition are required.

**Electronic supplementary material:**

The online version of this article (doi:10.1186/s12960-017-0195-2) contains supplementary material, which is available to authorized users.

## Background

Shortages of health workers are a critical public health issue in many countries. They prevent national health systems from meeting the needs of the population and achieving the sustainable development goal of universal health coverage (UHC) [1]. The International Labour Organization (ILO) estimated that in 2014, there was a global gap of 10.3 million health workers, for the achievement of UHC [2], and recent analysis from the World Health Organization (WHO) estimates a shortfall of 18 million health workers by 2030, which would prevent the achievement of the sustainable development goal (SDGs) [3]. In the 2013 Recife Declaration, global leaders declared their political commitment to tackling shortages in the health workforce [4]. This commitment is being carried forward with the WHO’s 2030 Global Strategy on Human Resources for Health [5], which was adopted at the 69th World Health Assembly in May 2016.

The size of a country’s health workforce is affected by both inflows and outflows, and it is essential that these labour market dynamics are well understood if countries are to be able to formulate effective workforce policies and strategies. Attrition—defined broadly as exits from the workforce, which can be due to emigration, voluntary exits (e.g. to other sectors of employment), illness, death or retirement—is an important element of outflows from the labour market and something that governments can directly influence by implementing strategies for health worker motivation and retention.

To address health worker shortages and plan effectively for the future, more focus needs to be dedicated to the issue of workforce attrition. High levels of attrition lead to a large loss of public resources spent on education and training of health workers [6]. Attrition also contributes to increased workload and worse working conditions for the remaining workforce, which in turn contributes to lower quality of care and worse health outcomes [7]. Exits from the workforce affect the projected supply of health workers that a country needs to meet population need for health care, making attrition a key component of workforce projection models [8].

The 2014 State of the World’s Midwifery (SoWMy) report [9], which focused on the sexual, reproductive maternal and newborn health (SRMNH) workforce, included the voluntary attrition rate as one of the 10 essential items needed for workforce planning. However, the SoWMy report found that these data are not readily available in many low- and middle-income countries. Often, countries with the largest attrition rates also have the lowest availability of data, as they lack reliable records which track attrition and migration of health workers [10].

One of the key components of attrition is out-migration, in particular from lower-income to higher-income countries, also known as ‘brain drain’. Driving this migration is the large demand for health workers in high-income countries due to ageing populations and the increasing burden of non-communicable diseases and health workers unsatisfied with low pay and lack of career progression opportunities in low-income countries [11, 12]. The region worst affected by this situation is sub-Saharan Africa, which also faces the most severe shortage of health workers [10].

It is hard to quantify the effects of brain drain. Data on health worker registration and country of origin in destination countries is often used to measure the magnitude of migration [13], but it is difficult to analyse the size of outflows from individual source countries. Furthermore, the consequences can be much more harmful than the numbers show: in low-income countries which have very few specialists in the health workforce, the migration of even a relatively small number of these can lead to great losses and eliminate educational opportunities [14]. It can also affect the overall institutional capacity of the health system to effectively develop and meet population needs [6]. Brain drain is considered an ethical concern as it exacerbates shortages of health workers in countries [6]. Global architecture such as the WHO Global Code of Practice on International Recruitment of Health Personnel [15] recognizes the severity of the issue and strives to negotiate a solution.

Health workforce attrition affects all countries in varying measures, although specific countries or regions may be hit hardest or by specific types of attrition. For example, the HIV/AIDS epidemic has led to higher rates of attrition due to illness, death or fear of infection in sub-Saharan Africa [14, 16]. However, attrition rates also vary within countries along multiple dimensions: for example, by sub-national regions, by location (urban or rural/remote) [17, 18], by type of health facility, by cadre or even by age of health worker. Retaining health workers in rural or remote areas is a particularly complex problem that affects all countries, including high-income countries that do not generally struggle with high rates of attrition in urban areas and numerous studies have proposed strategies to alleviate this problem [18].

Because of the need for accurate estimates of attrition rates and the lack of data in many countries, workforce projection models such as that used in SoWMy 2014 are (to a greater or lesser extent) reliant on assumptions and estimates until such time as data collection procedures can be improved. This study provides a comprehensive review of the available evidence on attrition rates and demonstrates the wide variability according to different types of health worker and different country contexts. It also explores how attrition is measured and studies are conducted, thereby drawing attention to the need for standard definitions and methodologies for measuring and monitoring this vital aspect of labour market dynamics.

## Methods

### Rapid literature review

We conducted a rapid review of studies published since 2005 on the attrition rates of health workers. Attrition was defined as any exit from the health workforce. We searched the PubMed, Web of Science and Human Resources for Health (HRH) Journal databases. The search expressions were adapted to each database based on the scope and relevance of the retrieved papers. The search expressions were the following:

- For PubMed: ((‘human resources for health’ OR ‘health workforce’ OR ‘health workers’ OR ‘health manpower’) AND (‘attrition rate’ OR exit OR losses OR ‘brain drain’ OR turnover OR retention))
- For Web of Science: ((‘human resources for health’ OR ‘health workforce’ OR ‘health workers’) AND (‘attrition rate’ OR exit OR losses OR ‘brain drain’ OR turnover OR retention))
- For HRH Journal: ((‘human resources for health’ OR ‘health workforce’ OR ‘health workers’) AND (‘attrition rate’ OR exit OR losses OR ‘brain drain’ OR wastage OR turnover OR migration))

The terms ‘turnover’ and ‘retention’ have different definitions from attrition. However, they are often used interchangeably in the literature, which is why they were included in the search expression: this increased the chance of collecting and extracting quantitative data or estimates of attrition.

In total, 841 papers were obtained from the PubMed search, of which 2 were excluded as duplicates; 417 papers were obtained from the HRH Journal search, of which 124 were excluded because they had already been identified via the PubMed search. From the Web of Science, 834 papers were retrieved, of which 190 were excluded due to duplication. Additionally, 17 papers were identified through expert consultation, of which 11 were duplicates. Thus, 1782 papers were identified.

**Fig. 1:**
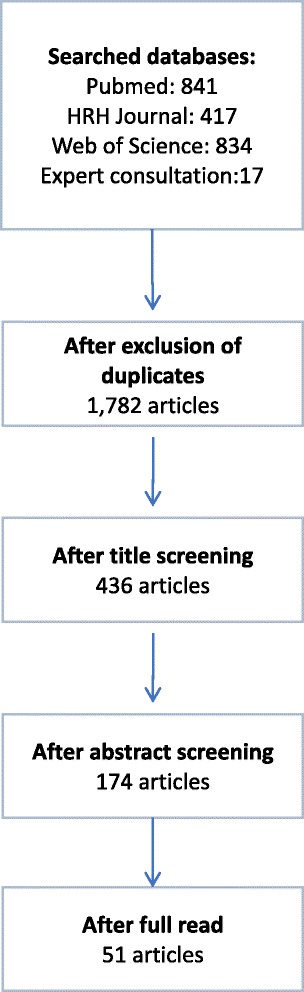
Diagram of the rapid review search

A title review was carried out by two researchers, who each reviewed half of the 891 titles; 436 papers were short-listed for abstract review based on the following criteria: specific to HRH and reference to the use of quantitative methods. The papers that mention the use of qualitative methods only were excluded. Upon this abstract review, 174 of the papers were selected to be read in full because the abstract included quantitative results about attrition (% of health workers leaving the workforce and attrition rates in a specific period of time). Studies reporting only retention rates or % of health workers retained were not considered. Of these 174 papers that were read in full, 123 were excluded because they focused rather on the intention to leave of health workers or on attrition from education than on actual estimation of attrition rates from the workforce. Although these are important losses which also need to be quantified, they occur at a different stage of the labour pipeline and therefore were beyond the scope of this study. Forty-five papers were selected based on these criteria, and additional 6 papers were identified by the authors through references from the selected papers which were also added to the review, making a total of 51 papers ﻿(Fig. 1).

A data extraction table was used to record information on objective of the study, countries involved, study setting (nationwide, sub-national), definition of attrition, cadres involved, type of attrition (total or voluntary), reasons for leaving and attrition rates.

Where possible, comparison of total and voluntary annual attrition rates was made. In a specific study [19], two different estimation processes were used to calculate the average of annual attrition for each cadre by country. The mean (unweighted) of the attrition provided by these two methods was then estimated and used in this study. Voluntary attrition was defined by the reasons to leave, when said reasons were presented in the studies.

### SoWMy 2014 dataset

In addition to the rapid review, we also conducted an analysis of voluntary attrition from the SRMNH workforce, using data from the State of the World’s Midwifery 2014 (SoWMy 2014) survey [9], as a large-scale of a multi-country approach. Seventy-three low- and middle-income countries provided data for the survey conducted in October 2013 to February 2014. The survey was completed by United Nations Population Fund (UNFPA) and WHO country offices in the participating countries, with validation of the data by stakeholders including Ministries of Health. In addition, six countries from the UNFPA Arab States Region completed the same survey between September 2014 and May 2015 for the follow-up report: Analysis of the Midwifery Workforce in selected Arab Countries [20], so these countries were also included in this analysis.

The 79 countries in the combined dataset were asked to respond to the following question for each of their SRMNH cadres in the workforce: ‘Approximately what percentage of this cadre left the workforce in the last year for reasons other than death or reaching statutory retirement age?’. This corresponds to the annual voluntary attrition rate. Each SRMNH cadre for which data were provided was classified by the researchers into a category from the International Standard Classification of Occupations (ISCO) [21], based on their roles and competencies. An analysis of the findings is provided in the ‘Results’ section of this paper.

## Results

### Rapid review

Of the 51 papers, 16 focus on the WHO African region [19, 22–36], 11 on the Americas [37–47], 10 on the Western Pacific [48–57] region, 5 on the Eastern Mediterranean region [58–62], 4 each on the European [63–66] and South-East Asia [67–70] regions and 1 on countries from different regions [71]. The studies were conducted in high-income countries (*n* = 19), low-income countries (*n* = 12), low-middle income countries (*n* = 9) and upper-middle countries (*n* = 7). In four studies, more than one country or region was included; therefore, these studies were not categorized by income group.

The objectives of the studies ranged from forecasting future needs of the health workforce to examining the current status of the workforce (availability), external and internal migration of the workforce, attrition within specific training or health programmes and retention of health workers (factors and levels).

Most studies took place at the national level (*n* = 29), using national data (from census, council registers or ministries of health). Sub-national studies (*n* = 21) focused on states, provinces, districts, rural and remote areas, health facilities and education institutions. This classification was not applied to one study as it involved a scoping review of the literature [71].

Only half of the studies provided a full definition of attrition. A small number of studies conducted a literature review of the definitions of attrition and/or used an international definition. The word ‘attrition’ was frequently used interchangeably with the terms ‘drop-outs’, ‘turnover’, ‘brain-drain’, ‘losses’, ‘premature departure’ and ‘separation’. Reasons for leaving the workforce were also used to define attrition, particularly migration. Others included retirement, resignation, dismissal or death.

Doctors, nurses (registered and enrolled nurses, licensed practical nurses, nurse assistants), midwives and community health workers (CHWs) were the cadres most often featured in the studies. Others included clinical officers, specialists, pharmaceutical staff, lab technicians, healthcare aides and allied health professionals.

Key reasons for attrition identified in the literature include low salaries, lack of access to professional development and further education, lack of effective supervision, weak regulatory environments, isolation (for health workers in rural or remote areas), poor working conditions (including facility conditions, lack of medical equipment and technology), stress or large caseloads and lack of motivation/low job satisfaction. In some countries, perceived lack of security is also a key factor in intentions to leave the health workforce.

The distinction between voluntary and total attrition was not clearly stated in most studies. Of the 51 papers, 29 provided information on total attrition, 18 on voluntary attrition and 4 on both.

Attrition rate estimates were provided for different periods of time, ranging from 3 months to 12 years. However, the annual attrition rate was the most common (*n* = 27) and the only comparable measure. Only one study [40] estimated attrition using full-time equivalents rather than headcounts.

Additional file 1 in the annex details all papers included in the review including the objectives, countries and settings, the definition of attrition provided and the total and voluntary attrition rate estimates by cadre.

Overall, the total annual attrition rate varied between 3 and 44% while the voluntary annual attrition rate varied between 0.3 and 28%. Table 1 shows how annual attrition varied by professional cadre, for doctors, nurses and midwives. Looking first at total attrition, out of the seven studies which included doctors, estimates of the total annual attrition rate varied from 1.7% in USA to 15% in Afghanistan. Out of the nine studies which included nurses, estimates of the total annual attrition rate varied from 4.9% (the average from several African countries) to 44.3% in New Zealand. Out of the four studies which included midwives, estimates of the total annual attrition rate varied from 4.5% in Zambia to 16% in Afghanistan. The two studies which included CHWs put forward estimates of the total annual attrition rate of 5% (in Afghanistan) and 22% (in Bangladesh). Estimates of voluntary attrition rates are considerably lower than estimates of total attrition rates. Within cadres, its variability is similar to total attrition. For doctors, the annual voluntary attrition rate ranged from 1% in Thailand to 10% in Romania and for nurses, between 1.4% in Zambia and 9.3% (an overall estimate for several European countries).

**Table 1:** Minimum and maximum estimates of total and voluntary annual attrition rates

	Doctors		Nurses		Midwives	
	Min	Max	Min	Max	Min	Max
*N* studies w/ total attrition^a^	7		9		4	
Total attrition rate	1.7%	15%	4.9%	44.3%	4.5%	16%
N studies w/ vol. attrition^a^	5		4		1	
Voluntary attrition rate	1%	10%	1.4%	9.3%	1.4%	–

^a^The total number of studies was 27, but some included data on more than one cadre, so the total number of studies in the table is greater than 27

Table 2 shows the annual attrition rates by income group (for the year of publication). For doctors, there is some indication that total annual attrition rates are higher in low-income countries than in high-income countries. Small sample sizes mean that it is more difficult to distinguish a pattern for other cadres and for voluntary attrition.

**Table 2:** Minimum and maximum estimates of total and voluntary annual attrition rates by country income group

	Total			Voluntary		
Income group	Doctors	Nurses	Midwives	Doctors	Nurses	Midwives
High	1.7%	4.5–17.3%	–	–	6–9.3%	–
Middle	9.8%	5.3–44.3%	4.5%	1–10%	1.7%	1.4%
Low	15%	14%	9–16%	3.7%	7.6%	–

At the sub-national level, the availability of annual rates is low, which limits comparability.

Two studies analysed differences between sub-national regions: one provided separate attrition rates for all [61] and the other provided estimates from regional (non-urbanized areas), rural and remote levels [54]. The data show higher attrition rates in remote areas compared with rural areas (30.2 and 18.7% respectively).

A few studies considered how attrition rates vary by type of health facility as well as professional cadre. These seem to indicate variability within the same cadre according to the type of health facility to which the health workers are deployed, but there are insufficient data to draw general conclusions about how the rate of attrition varies by type of health facility, except to note that total attrition seems to vary more by facility type than voluntary attrition.

Attrition due to migration was addressed exclusively in 11 studies (2 included both internal and external migration and 9 external migration only) [31, 32]. Nearly half involved physicians, and only two provided annual rates: 3.7% [31] and 10% [65].

### SoWMy 2014 dataset

Of the 79 countries in the combined dataset, 49 provided some data on voluntary attrition rates of their SRMNH cadres, i.e. 30 countries could not provide even an estimate (see Additional file 2 for SoWMy Dataset on attrition). Data on attrition were provided for 166 unique SRMNH worker cadres, which represents a response rate of 40.5% from the all cadres in the dataset. These cadres were mapped against the corresponding ISCO-08 classification, yielding the results presented in Table 3 below.

**Table 3:** Average voluntary attrition rates by type of health worker (headcounts), SoWMy 2014 survey

Type of health worker (ISCO Classification)	*N* (cadres)	Average (mean) annual voluntary attrition rate (%)	Standard deviation	Median	Min	Max
Generalist medical practitioners	33	8.8	11.7	4.0	0	45
Specialist medical practitioners (ob/gyns)	36	4.5	7.8	1.0	0	30
Nursing professionals	10	7.2	6.5	10.0	0	20
Midwifery professionals	53	8.2	10.5	2.5	0	45
Associate nursing professionals	5	4.6	7.8	0	0	20
Associate midwifery professionals	24	5.9	6.5	3.0	0	23
Paramedical practitioners and medical assistants	5	0.4	0.8	0	0	1.9
Total	166	6.8	9.5	2	0	45

In SoWMy, the highest attrition rates were recorded for generalist physicians and midwives. Conversely, specialist medical practitioners (in the survey, these were obstetrician/gynaecologists) had the lowest rates of attrition (excluding paramedical practitioners and medical assistants, which had a very small sample size). It should be noted, however, that attrition rates were very widely spread, with wide ranges and large standard deviations. When comparing with the result ranges obtained in the rapid review, the SoWMy results show wider variation overall.

## Limitations

The rapid review included studies which defined attrition in different ways and measured it over different time frames, which has reduced the comparability of the data and prevented more sophisticated analyses. Here we limit ourselves to presenting and comparing the estimates of attrition which were provided as annual rates. The involvement of different settings and regions has also increased the diversity of methodologies and methods of measurement, which also limits the direct comparison of the data. In addition, gender variations in voluntary attrition rates were not considered, despite the likelihood that gender inequities and discrimination contributes to its variability. Neither was the study able to differentiate between public sector and private sector health workers, despite the likelihood that attrition rates vary between the two. Furthermore, none of the studies included in the rapid review provided estimates of uncertainty around the reported results, which would have been a useful addition to this study. A meta-analysis could provide further insight on this subject.

Most countries participating in the SoWMy 2014 study were unable to provide empirical data about voluntary attrition rates and instead had to rely on expert estimates. Although these were approved by ministries of health, they may still be inaccurate. They also represent a relatively resource-intensive, one-off approach, which not only illustrates that there is a scope to develop better estimates of attrition but also highlights that the need is to systematize data collection and support regular analysis and reporting.

## Discussion

This study identified and reviewed a number of papers that directly or indirectly estimate health workforce attrition rates. In its original design, it aimed to examine only voluntary attrition, defined as exits from the workforce for reasons other than death or retirement. However, due to the low number of studies that made a distinction between voluntary and other forms of attrition, it was decided to expand the inclusion criteria to all forms of attrition.

Overall, there is lack of data and such data that exist are not particularly comparable. This is also supported by the multi-country analysis of the SoWMy 2014 data, where even in the case of data collected and validated with the support of national ministries of health for a single multi-country study using a standard approach, attrition rates were only estimated by 62% of participating countries and for only 40% of the SRMNH cadres included in the survey.

The current study highlights the marked variation in reported attrition rates, between countries, within countries and across time. Accurate attrition rates are vital to labour market analysis, workforce planning and assumptions on future supply requirements. A large number of studies on attrition are conducted as surveys amongst health workers of intentions to leave [10, 72–76]. Although these have great value in exploring the causes of attrition and measuring the overall job satisfaction or motivation of the health workforce, they may not provide an accurate estimate of real attrition rates, as not all those who intend to leave may have the ability to carry out this intention. There is an urgent need to agree definitions and support both more intensive research-based examinations of the reasons for variations in attrition rates, as well as support to systematize analysis and reporting. This is particularly important for GPs and midwives, due to their direct impact on meeting the SDG3 targets, namely UHC, universal access to SRMNH and expansion of primary health care [77].

Moreover, this study also suggests that attrition rate estimates are influenced by the purpose and type of study (design, etc.), i.e. results vary according to the data collection method, if it involves secondary sources, or uses surveys to the facilities or health workers, if all cadres are involved or only one, if intends to evaluate turnover from a specific programme in a short period of time, amongst other study designs. Attrition is an essential piece of information to adequately plan and manage the health workforce in any country [78]; however, data on attrition are not yet routinely collected. Many studies highlight the lack of specific data, using census or the professional council datasets—which may not always provide accurate numbers for voluntary attrition. This imposes many difficulties in the measurement of attrition rates, leading to a wide variety of approaches, which cannot always be generalized to countries other than the one under study [79]. To accurately study and measure attrition, cohort studies are the best approach but these can be relatively complex to establish and can be resource intensive to implement systematically. There is also the large-scale multi-country study approach, like SoWMy, which can provide a large dataset of comparable data, but it is not the most sustainable approach for countries and international partners. Moreover, even in the SoWMy study, countries were still unable to provide accurate estimates of their attrition levels, relying on less accurate techniques like expert consultation. This points to the need for countries to embed an agreed measure of attrition in a minimum dataset.

An additional challenge is posed by the lack of an internationally recognized definition of attrition and types of attrition (voluntary, involuntary, etc.), which leads to a great variability in terminology and interchangeable use of terms that increase diversity and further reduce comparability [47]. In addition, health data records are generally poor and/or of low quality for this type of data. This is a pervasive issue across many different countries and levels of the health system. Even in more sophisticated workforce forecasting methodologies, mostly undertaken in high- and upper-middle income countries [45, 80], assumptions and additional steps are used to obtain estimates of attrition rates.

At present, there is a global call to obtain more and better data, in particular HRH data, with the momentum driven by the new global strategy and the SDGs. The WHO has developed a minimum dataset which lists the essential data items, including attrition levels, required from each country for effective HRH planning [81]. This is expected to increase comparability across countries and regions, as well as, at a long-term basis, to allow exchange/sharing of similar data across regions and countries [82].

The findings of this review reinforce the importance of disaggregating information, be it geographically, by health worker cadre, or by level of care, as attrition rates vary at all these different levels [83]. This suggests that a national system of records is critical, as voluntary attrition represents a substantial, if variable, percentage of total attrition and is bound to impact on HRH planning processes. It is reasonable to suppose that involuntary attrition rates may have relatively less variation between institutions within countries (due to relative uniformity in statutory retirement ages and life expectancy), but the potential or variation in voluntary attrition makes it a critical focus for analysis. There is a need for an approach where each health worker has a unique ID number that allows tracking of movement and changes in employment status. It becomes more easy to collect such standardized data using the technology now widely available (e.g. mobile phones, internet) [84, 85]. One route for improvement would be to use the application of the WHO Minimum Dataset (MDS) [81] as a mechanism to support consistency in collection of supply data on the health workforce.

Finally, this study indicates that in terms of voluntary attrition, the greatest focus in the literature, particularly from low and middle income countries, is on brain drain or migration to other countries. As such, the other forms of voluntary attrition may be underestimated, in particular the internal migration of health workers, often related to a preference for urban areas [79]. Equitable access to health and UHC may be compromised if countries are unable to accurately identify where the gaps are. The literature is scarce when it comes to quantifying the size of these losses; therefore, further attention is required to this subject.

## Conclusions

This paper has examined key issues associated with definitions of, and research on, health workforce attrition. Attrition may also be an indicator of one or more of a variety of work-related problems, such as overwork, poor job satisfaction, uncompetitive pay and career opportunities and lack of effective supervision. One main finding in this paper is the lack of a standardized definition of attrition, both in routine reporting, and more detailed research-based analysis. This represents a major constraint on developing a better understanding of the extent to which the levels and types of attrition vary in different organizations and therefore also limit the scope to use attrition data as a tracer or indicator of other factors and as a source of comparison between organizations and systems.

One approach which would enable a clearer evidence base to emerge on attrition would be to support a standardization of definitions and methods of measuring attrition. This in turn would then enable a clearer picture to emerge at organizational level about the real scope for policy and management intervention to reduce voluntary attrition and greater scope for comparison across organizations to identify relatively ‘low’ and ‘high’ attrition sites which may be worthy of more in depth analysis. Furthermore, attrition data could be disaggregated by reasons for leaving and by geography, health facility type and cadres to make sure that variability in these areas is captured, thus allowing countries to make strategically intelligent decisions about current and future workforce education, deployment and management. One option is to use the application of the WHO MDS [81] to frame a dialogue that focuses on the benefits for attrition-related data collection.

## Additional files

Additional file 1: Annex: **Table S1.** Summary of the papers included in the review. (DOC 120 kb)
Additional file 2: SoWMy Dataset for Attrition. (XLSX 14 kb)

## Abbreviations

CHW: Community health workers
GPs: General practitioners
HRH: Human Resources for Health
ILO: International Labour Organization
ISCO: International System of Classification of Occupations
SoWMy: State of the World’s Midwifery
SRMNH: Sexual, reproductive, maternal and neonatal health
UHC: Universal health coverage
UNFPA: United Nations Population Fund
WHO: World Health Organization
