# Supplementary material for: A rapid review of the rate of attrition from the health workforce
Source: Hum Resour Health. 2017 Mar 1;15:21. doi: 10.1186/s12960-017-0195-2 (PMC5333422; doi:10.1186/s12960-017-0195-2)
Supplement: Additional file 1: — Annex: Table S1. Summary of the papers included in the review. (DOC 120 kb) [file 12960_2017_195_MOESM1_ESM.doc]

**Annex: Table S1: Summary of the papers included in the review**

| **Authors/ Year of publication** | **Title** | **Objective(s)** | **Countries involved/ setting** | **Income group** | **Definition of attrition** | **Cadres** | |
| --- | --- | --- | --- | --- | --- | --- | --- |
|  |  |  |  |  |  | **Total attrition rate*** | **Voluntary attrition rate*** |
| Abbey M, et al./2014 | Factors related to retention of community health workers in a trial on community-based management of fever in children under 5 years in the Dangme West District of Ghana. | To examine the attrition rate among CHWs who participated in a cluster randomised controlled trial (RCT) on community management of fever in children under-5 in the Dangme West District of Ghana and the factors contributing to the retention of CHWs. | Ghana/ Sub-national (district in a specific intervention) | Low- middle | Describes the loss of CHWs to the intervention from the time of deployment until the end of the project. | CHW: 21.3% (over 30 months) | - |
| Adelberger A, Neely-Smith S, Hagopian A./2010 | Estimating nurse migration from the Bahamas between 1994 and 2005: An exploratory descriptive study using a social network identification methodology | To collect primary data on the migration patterns of Bahamian nurses who had registered as nurses during the period 1994 and 2005. | Bahamas/ Nationwide | High | ‘Brain drain’: to move to countries that offer more attractive pay, better quality of life and more educational opportunities. | - | Nurses migration: 6.0% (over 12 years) |
| Alam K, Oliveras E./  2014 | Retention of female volunteer community health workers in Dhaka urban slums: a prospective cohort study. | To understand the factors associated with retention of volunteer CHWs. | Bangladesh/ Sub-national (urban slums) | Low- middle | Drop-outs (not clearly defined). | CHW: 22.0% | - |
| Ansah J, De Korne, D, et al. /  2015 | Future requirements for and supply of ophthalmologists for an aging population in Singapore. | To forecast ophthalmology workforce and training requirements for Singapore up to the year 2040. | Singapore/ Nationwide | High | Blended value of retirements, deaths, and resignations. | Ophthalmologists: 3% | - |
| Bloom J, Duckett S,  Robertson A./  2012 | Development of an interactive model for planning the care workforce for Alberta: case study. | To develop a forecasting tool to test the effect of different assumptions (for instance about vacancy rates or retirement) and different policy choices for Registered Nurses, Licensed Practical Nurses and health care aides. | Canada/ Sub-national (province) | High | Separation rate or staff turnover (not clearly defined). | Registered nurses: 4.5%  Licensed practical nurses: 5.8%  Healthcare aids: 7.6% | - |
| Bukach A M, Ejaz F K, et al. /2015 | Turnover among Community Mental Health Workers in Ohio. | To examine turnover of community mental health workers in 42 randomly selected mental health agencies in Ohio. | USA/  Sub-national (state) | High | Workers who left the mental health agency voluntarily (quit) or involuntarily (were terminated or fired), as well as workers who moved laterally to a different position or were promoted to another position. | Community mental health workers: 26.0% | Community mental health workers: 17.4% |
| Burnham G M, Lafta R, Doocy S./2009 | Doctors leaving 12 tertiary hospitals in Iraq, 2004-2007. | To explore how migration of specialists has affected staffing of tertiary hospitals, we reviewed hospital personnel records in twelve tertiary hospitals from 2004 through late 2007. | Iraq/  Sub-national (Health facilities – 12 tertiary hospitals) | Low- middle | n/a | Physicians: 46% (over 3 years)  Of which:  74% moving transfer  12% retirement  7% escaping threats  6% violence and threats  1% death | - |
| Chankova S, Sulzbach S./2006 | Zambia Health Services and Systems Program. Occasional Paper Series. Human Resources for Health, Number 1 | The paper presents an overview of the current HRH situation in Zambia, provides comparisons with other countries in the region. (1st paper of a series) | Zambia Cote de Ivoire Ethiopia/ Nationwide | n/a | To leave the country to take better-paid health positions abroad or simply exit from the medical profession for more lucrative positions; and HIV/AIDS impact. | Physicians, nurses and pharmacists  All: 5.4%  Of which:  38% death  32% resignation  12% dismissal  10% retirement  Physicians:  9.8% Zambia  9.0% Côte d'Ivoire 9.6% Ethiopia  Nurses:  5.3% Zambia  7.0% Côte d'Ivoire  3.2% Ethiopia  Pharmacists : 4.2% | - |
| Chankova S, Muchiri S, Kombe G./  2009 | Health workforce attrition in the public sector in Kenya: a look at the reasons. | To look into the status of attrition rates and the proportion of attrition due to retirement, resignation or death among doctors, clinical officers, nurses and laboratory and pharmacy specialists in surveyed facilities. | Kenya /Nationwide (provincial hospitals, district hospitals, health centres) | Low | Number of health workers who left the facility between mid-2004 and mid-2005, divided by the number of health workers who were employed by the facility in mid-2004. Reasons for leaving included resignation, retirement, death and transfer to another health facility within the public sector. | All health professionals:  Provincial hospitals (PH): 4%  District hospitals (DH): 3%  Health centres (HC): 5%  Physicians:  PH: 7.5%  DH: 3.7%  Registered nurses:  PH: 6.2%  DH: 2.4%  HC: 2.3%  Enrolled nurses:  PH: 2.9%  DH: 3.0%  HC: 2.0%  Clinical officers:  PH: 1.5%  DH: 1.6%  HC: 3.0%  Lab staff:  PH: 1.5%  DH: 6.5%  HC: 8.1%  Pharmaceutical staff:  PH: 10.0%  DH: 13.9%  HC: 14.3% | All health professionals:  PH: 1.6%  DH: 1.1%  HC: 1.3%  Registered nurses:  PH/DH: 0.7%  HC: 0.8%  Enrolled nurses (HC): 0.3%  Clinical officers: 1.3%  Lab staff: 1.6%  Pharmaceutical staff  PH/DH: 0.8%  HC:3.6% |
| Chisholm M, Russell D, Humphreys J./2011 | Measuring rural allied health workforce turnover and retention: what are the patterns, determinants and costs? | To measure variations in patterns of turn- over and retention, determinants of turnover, and costs of recruitment of allied health professionals in rural areas. | Australia/ Sub-national | High | Annual turnover rate: the proportion of the workforce that left during each calendar year. | Allied health professionals (dietician, occupational therapist, physiotherapist, podiatrist, psychologist, social worker, speech pathologist): 26%  Regional: 18.7%  Rural: 25.4%  Remote: 30.2% | - |
| Chiscop E./  2013 | Tackling the brain drain from Romania's health workforce: problem analysis and policy recommendations. | To identify and define ‘brain drain’ as one of the main problems of the Romanian health system. The final objective of this paper is to urge the ministry to review its policies and to submit a national strategic plan to tackle the issue | Romania/ Nationwide | Upper-middle | Only migration to Western Europe. | - | Physicians: 10% (over 3 years) |
| De Silva A, et al./  2013 | Migration of Sri Lankan medical specialists. | To describe the migration of medical specialists from Sri Lanka and to discuss the successes and failures of strategies to retain them. | Sri Lanka/ Nationwide | Low-middle | Migration (% of total workforce in 2010 that migrated 2006-2009). | - | Specialists migration:  Internal medicine: 8.2%  General surgery: 7.0%  Anaesthesia: 12.2%  Psychiatry: 27.8%  Other specialities: 5.2% |
| Donoghue C./2010 | Nursing Home Staff Turnover and Retention: An Analysis of National Level Data. | To provide national estimates of turnover and retention for registered nurses, licensed practical nurses, and certified nursing assistants in nursing homes, and to examine the associations between management tenure, organizational characteristics, local economic conditions, turnover, and retention. | USA/ Nationwide | High | Turnover: nurses that quit in full-time equivalents. | Nurses:  Certified nursing assistants: 74.5%  Registered nurses: 56.1%  Licensed practical nurses: 51.0% | - |
| Doiron D, Hall J,  Jones G./  2008 | Is there a crisis in nursing retention in New South Wales? | To conduct a systematic analysis of trends in nursing retention rates over time | Australia/ Sub-national (state) | High | Retention: continuing to work in the state's nursing workforce | 1994-95  Registered nurses: 16.1%  Enrolled nurses: 22.9%  Total: 17.3%  1999-2000  Registered nurses: 15.1%  Enrolled nurses: 19.6%  Total: 15.9% | - |
| El-Jardali F, Dumit N, Jamal D, Mouro G./2008 | Migration of Lebanese nurses: a questionnaire survey and secondary data analysis. | To provide an evidence base for understanding the incidence of nurse migration out of Lebanon, its magnitude and reasons. | Lebanon/ Nationwide | Upper-middle | Migration: nurses working abroad. | - | Nurses migration: 18.9% (over 6 years) |
| Faqir M, Zainullah P, et al./ 2015 | Availability and distribution of human resources for provision of comprehensive emergency obstetric and newborn care in Afghanistan: a cross-sectional study. | To assess the availability and distribution of human resources for round-the-clock comprehensive emergency obstetric and newborn care service provision in secure areas of Afghanistan in order to inform policy and program planning. | Afghanistan/ Nationwide | Low | Turnover: includes all health workers leaving their post in 2009, whether due to transfers, dismissal, retirement, or other reasons. | Physicians: Total: 15%  HC: 40.0%  DH: 13.0%  PH: 15.0%  RH: 0  SpH: 1.0%  Nurses: Total: 14.0%  HC: 18.0%  DH: 12.0%  PH: 12.0%  RH: 20.0%  SpH: 20.0%  Midwives: Total: 16.0%  HC: 24.0%  DH: 19.0%  PH: 11.0%  RH: 8.0%  SpH: 12.0%    Anaesthetist: 16.0%  Ob/gyn: 28.0%  Pediatrician: 18.0%  General surgeon: 27.0%  Pharmacist: 9.0%  Lab technician: 13.0% | - |
| Galaty D, Banda P./  2005 | Resources crisis in the Zambian health system: A call for urgent action. | To analyze the human resource requirements associated with scaling up the provision of ART, PMTCT, and VCT services in the public health sector, and estimate future national human resource needs based on current attrition, graduation, and retention. | Zambia/ Nationwide | Low-middle | n/a | All health workers (physicians, nurses, pharmacists, lab technicians): 5.4%  Physicians: 9.8%  Nurses: 5.3%  Pharmacists: 4.2%  Lab technicians: 3.5% | - |
| Ginde AA, Sullivan AF, Camargo CA./  2010 | Attrition from emergency medicine clinical practice in the United States | To estimate the annual attrition from emergency medicine clinical practice. | USA/ Nationwide | High | Not being active in emergency medicine clinical practice. This includes active in non–emergency medicine clinical practice, retired, administration, teaching, research, and not active for other reasons. | Physicians (emergency physicians only): 1.7% | - |
| Hailemichael Y, Jira C, Girma B, Tushune K./ 2010 | Health workforce deployment, attrition and density in East wollega zone, Western ethiopia. | To assess health workforce density, deployment and attrition. | Ethiopia/ Sub-national | Low | Number of health workforce who drop out their job in specified period of time as numerator and available health workforce at the end of the same period as denominator. | All health cadres (only health professionals):  Before decentralization (2000-2002): 10.2%  After decentralization (2003-2005): 18.8%  2000-2005  Specialists and GPs: 47.2%  CHWs ( primary health workers, primary midwife and health extension workers): 1.1% | - |
| Hasselhorn HM, Müller BH, Tackenberg P./2005 | NEXT Scientific Report | To investigate the reasons, circumstances and consequences surrounding premature departure from the nursing profession in Europe. | European/ Nationwide (Belgium, Finland, France, Germany, Great Britain, Italy, the Netherlands, Poland, Sweden and Slovakia) | High | Premature departure from the profession - those who actually have left their institution during the 12 months period following the assessment. | - | Nurses: 9.3% |
| Hewko SJ, Cooper SL, Huynh H, Spiwek TL, Carleton HL, Reid S, et al./2015 | Invisible no more: a scoping review of the health care aide workforce literature. | To evaluate the breadth and depth of the HCA workforce literature. | Global | n/a | Turnover (not clearly defined). | Health care aide:  Minimum: 59.4%  Maximum: 170.5% | - |
| Joyce CM, McNeil JJ./2006 | Participation in the workforce by Australian medical graduates | To investigate workforce participation patterns among Australian medical graduates and the extent of cohort differences in these patterns. | Australia/ Nationwide | High | Proportion remaining in the Australian medical workforce, based on number of years after graduation between 1980 and 1995 (2, 5, 8, 10 and 15). | Physicians, by number of years after graduation:  2 yrs: 4%  5 yrs: 10%  8 yrs: 11%  10 yrs: 15%  15 yrs: 12% | - |
| Kaushik M, Jaiswal A, Shah N, Mahal A./2008 | High-end physician migration from India. | To examine the relation between the quality of physicians and migration among alumni of All India Institute of Medical Sciences (AIIMS), | India/ Sub-national (1 medical school) | Low-middle | Migration (not clearly defined). | - | Physicians Migration:  54% (of graduates 1989-2000) |
| Kinfu Y, Dal Poz MR, Mercer H, Evans DB./2009 | The health worker shortage in Africa: are enough physicians and nurses being trained? | To estimate systematically the inflow and outflow of health workers in Africa and examine whether current levels of pre-service training in the region suffice to address this serious problem, taking into account population increases and attrition of health workers due to premature death, retirement, resignation and dismissal. | African countries/ Nationwide | n/a | Outflows: includes premature deaths among health workers, dismissals, resignations (e.g. to migrate or change career) and retirement. | Physicians (total for 12 African countries): 5.2%  Central African Republic: 5.8%  Côte d’Ivoire: 5.6%  DR Congo: 5.2%  Ethiopia: 5.1%  Kenya: 5.1%  Liberia: 6.8%  Madagascar: 4.9%  Rwanda: 5.1%  Sierra Leone: 7.2%  Uganda: 4.7%  United Republic of Tanzania: 5.2%  Zambia: 5.2%  Nurses and midwives (total for 12 African countries): 4.9%  Central African Republic: 4.9%  Côte d’Ivoire: 4.4%  DR  Congo: 4.8%  Ethiopia: 4.7%  Kenya: 5.0%  Liberia: 4.8%  Madagascar: 5.4%  Rwanda: 4.0%  Sierra Leone: 5.0%  Uganda: 4.9%  United Republic of Tanzania: 5.2%  Zambia: 4.8% | - |
| Kober K, Van Damme W./2006 | Public sector nurses in Swaziland: can the downturn be reversed? | To describe the current HRH situation in the public sector in Swaziland. | Swaziland/ Nationwide | Low-middle | Outflows: retirement, retirement/death due to HIV/AIDS and emigration (2010). | Nurses: 29.3% | - |
| Ludwick T, Brenner JL, Kyomuhangi T, Wotton KA, Kabakyenga JK./2014 | Poor retention does not have to be the rule: retention of volunteer community health workers in Uganda. | To analyse the retention and motivation of volunteer community health workers trained by HCU. | Uganda/ Sub-national (rural) | Low | No longer in service, for: work load, moved from village, business/employment, died, separation/divorce, health/family issues, opposed by husband, unknown, fired, further studies. | CHW: 13.0% (over 5 years) | CHW:11.0% (over 5 years) |
| Mandeville KL, Ulaya G, Lagarde M, Gwesele L, Dzowela T, Hanson K, et al./2015 | Early career retention of Malawian medical graduates: a retrospective cohort study. | To examine the retention of recent medical graduates within Malawi and the public sector. | Malawi/ Nationwide | Low | Migration (not clearly defined). | - | Physicians migration: 13.0% (over 7 years) |
| Mansoor GF, Hashemy P, Gohar F, Wood ME, Ayoubi SF, Todd CS./2013 | Midwifery retention and coverage and impact on service utilisation in Afghanistan | To measure the rate of and determine factors associated with community midwifery education (CME) graduate retention in public sector health care in Afghanistan | Afghanistan/ Sub-national (various provinces) | Low | Retention: community midwifery education graduates employed at any public sector facility. | Midwives:  By province:  30.0% Farah  24.0% Faryab  39.0% Ghazni  16.0% Hirat  48.0% Jawzjan  72.0% Khost  31.0% Laghman  43.0% Paktia  33.0% Samangan  32.0% Saripul  25.0% Takhar | - |
| Meyer D, Raffle H, Ware LJ./2014 | The first year: employment patterns and job perceptions of nursing assistants in a rural setting. | To follow rural certified nursing assistants (CNAs) (n = 123) in the United States for 1 year post-training to identify retention and turnover issues in the long-term care (LTC) setting by exploring the CNAs’ perceptions of LTC work experience. | USA/ Sub-national | High | % of nurses who, at 1 year post-training, had worked in long-term care and then left. | Nursing assistants: 30.9% | - |
| Najafizada S,, Labonte R, Bourgeault IL./2014 | Community health workers of Afghanistan: a qualitative study of a national program. | To describe the CHW program in Afghanistan, explore the gender dynamics of the workforce, and identify facilitators and challenges to the program. | Afghanistan/ Nationwide | Low | Drop-outs (not clearly defined). | CHW: 5.0% | - |
| North N, Leung W,  Ashton T, et al./2013 | Nurse turnover in New Zealand: costs and relationships with staffing practises and patient outcomes | To develop and pilot test an new survey to assess Vermont’s health care workforce in a more reliable and valid manner, including new ways to measure the state’s need and demand for various health care workers. | New Zealand/ Nationwide | High | Nurse turnover: the process by which nurses leave or transfer from their primary employment position in health services (including retirement, resignation and internal transfer). | Nurses: 44.3% | - |
| North N, Leung W, LeeR./2014 | New graduate separations from New Zealand's nursing workforce in the first 5 years after registration: a retrospective cohort analysis of a national administrative data set 2005-2010 | To describe workforce separation rates and its relationship with demographic and work characteristics in the 2005 new graduate cohort’s first 5 years as practising registered nurses in New Zealand (NZ). | New Zealand/ Nationwide | High | Separation rate: % no longer practising nursing in NZ. (We used the Nursing Council definition of ‘practising’ as ‘eligible to practise’ even if not currently active, e.g. looking for work.) | Registered nurses: 25.6% (over 5 years)  2005-06: 17.8%  2006-07: 0.9%  2007-08: 1.6%  2008-09: 3.8%  2009-10: 1.5% | - |
| Olang’o CO, Nyamongo IK, Aagaard-Hansen J./2010 | Staff attrition among community health workers in home-based care programmes for people living with HIV and AIDS in western Kenya. | To examine trends and underlying causes of attrition among volunteer community health workers in home-based care for people living with HIV and AIDS in western Kenya. | Kenya/ Sub-national | Low | Drop-outs (not clearly defined). | CHW: 33.3% (over 11 months) | - |
| Oman KM, Moulds R, Usher K./2009 | Specialist training in Fiji: why do graduates migrate, and why do they remain? A qualitative study. | To explore reasons for losses of graduates to overseas migration and to the local private sector from the Fiji public workforce. | Fiji/ Nationwide | n/a | Migration (not clearly defined). | - | Specialist migration: 30.3% (over 7 years) |
| Oman K, Rodgers E, Usher K, Moulds R./2012 | Scaling up specialist training in developing countries: lessons learned from the first 12 years of regional postgraduate training in Fiji -- a case study. | To describe the evolution of postgraduate specialist training in the Pacific over the past 12 years. | Fiji/ Nationwide | Upper-middle | Migration (not clearly defined). | - | Specialist migration: 29.2% (over 12 years) |
| Pagaiya N, Kongkam L, Sriratana S./2015 | Rural retention of doctors graduating from the rural medical education project to increase rural doctors in Thailand: a cohort study. | To examined the impact of ‘Collaborative Project to Increase Rural Doctors (CPIRD)’ in relation to doctor retention in rural areas and public health service. | Thailand/ Nationwide | Upper-middle | Public retention: those whose place of work was at any hospital under the Ministry of Health as in July 2011. | Physicians: 45.9% (over 8 years) | - |
| Page S, Willey K./2007 | Workforce development: planning what you need starts with knowing what you have. | To report on the approach to workforce development taken by North Coast Area Health Service (NCAHS) in NSW, Australia. | Australia/ Sub-national | High | Separation rate (not clearly defined). | Nursing; allied health; medical; health management; administration; operations; dental; trades/engineering; aboriginal health; professional/technical: 7.9% | - |
| Park SH, Boyle DK, Bergquist-Beringer S, Staggs VS, Dunton NE./2014 | Concurrent and Lagged Effects of Registered Nurse Turnover and Staffing on Unit-Acquired Pressure Ulcers | To examine the concurrent and lagged effects of registered nurse (RN) turnover on unit-acquired pressure ulcer rates and whether RN staffing mediated the effects. | USA/ Nationwide | High | Turnover: leaving the position for any reason. | Nurses: 5.8% (quarterly rate) | - |
| Reinier, Kyndaron Palumbo, et al./2005 | Measuring the nursing workforce: clarifying the definitions. | To develop and pilot test a new survey to assess Vermont’s health care workforce in a more reliable and valid manner, including new ways to measure the state’s need and demand for various health care workers. The goals of this research were to (1) conduct a literature review to identify published definitions of vacancy and turnover rates, (2) examine the use of vacancy and turnover rates in practice, (3) explore measures of perceived need for nurses, (4) pilot a new survey designed for workforce data collection, and (5) recommend an improved methodology for state wide collection of routine nursing workforce data. | USA/ Sub-national (state) | High | Turnover: the number of individuals or positions leaving in a particular time period divided by the total number of individuals or RN positions during that period, expressed as a percentage. For annual turnover rates, the average number of employees for the year was estimated by adding the number of employees at the beginning and end of the year and dividing by two. | - | Nurses:  Hospitals: 13.0%  Home health agencies: 24.0%  Long term care facilities: 35.0%  Physician’s offices: 8.0% |
| Rouleau D, Fournier P, Philibert A, Mbengue B, DumontA./2012 | The effects of midwives' job satisfaction on burnout, intention to quit and turnover: a longitudinal study in Senegal. | To explore the job satisfaction, burnout, intention to quit, turnover rates and destinations of a national sample of midwives in Senegal. Its longitudinal design also allows for an investigation of which facets of job satisfaction are associated with three purported effects: burnout, job search activities and turnover. | Senegal/ Nationwide | Low | Turnover: the process by which staff leave or transfer within the health care system. Can be voluntary, e.g leaving to find another job;non-voluntary, e.g.. being dismissed;  Intra-sector, e.g. from a rural to urban public hospital;  Inter-sector, e.g. from public to private, or leaving the health care system altogether. | Midwives: 9.0% | - |
| Rudman A, Omne-Ponten M, Wallin L, Gustavsson P J./ 2010 | Monitoring the newly qualified nurses in Sweden: the Longitudinal Analysis of Nursing Education (LANE) study. | To present the LANE study, including: to estimate representativeness and analyse response rates over time, and also to describe common career pathways (including intention to leave nursing and occupational turnover) and life transitions during the first years of working life. | Sweden/ Nationwide | High | Turnover (not clearly defined). | - | Nurses: at least 2.0% |
| Sherr K, et al./2012 | Brain drain and health workforce distortions in Mozambique | To documents the extent of both internal and external physician migration from the public sector in Mozambique among physicians qualifying from medical school between 1980–2006. | Mozambique/ Nationwide | Low | External migration: physicians living and working outside of Mozambique  Internal migration: physicians working inside Mozambique, but no longer paid through the public sector. | - | Physicians migration: 3.7% |
| Suphanchaimat R, WisaijohnT, Thammathacharee N, Tangcharoensathien V./ 2013 | Projecting Thailand physician supplies between 2012 and 2030: application of cohort approaches. | To forecasts physician supply between 2012 and 2030 based on future production capacity and losses from the profession, and assesses whether, and if so when, the projected supplies will meet the respective goals of one doctor per 1,500 population as proposed by the 7th National Conference on Medical Education in 2001, and one per 1,800 population as proposed by the Ministry of Public Health (MoPH) in 2004. | Thailand/ Nationwide | Upper-middle | n/a | - | Physicians: 1.0% |
| Tomblin Murphy G, MacKenzieA, Guy-Walker J, Walker C./ 2014 | Needs-based human resources for health planning in Jamaica: using simulation modelling to inform policy options for pharmacists in the public sector | To describe the development and application of a needs-based HRH simulation model for pharmacists in the SERHA | Jamaica/ Nationwide | Upper-middle | Exits due to retirement, relocation, or death. | Pharmacists: 5.0% |  |
| Tomblin Murphy G./2012 | Eliminating the shortage of registered nurses in Canada: an exercise in applied needs-based planning. | To demonstrate the application of the needs-based approach to the problem of the shortage of registered nurses in Canada. | Canada/ Nationwide | High | n/a | Registered nurses:  Aged 35-49: 2.0%  Aged over 60: 11.0% | - |
| Tjoa A, et al./2010 | Meeting human resources for health staffing goals by 2018: a quantitative analysis of policy options in Zambia | To present single variable and multiple variable scenario analyses of the supply of health workers in a model that uses health workforce to population ratios to understand minimum staffing requirements in Zambia | Zambia/ Nationwide | Low-middle | Voluntary attrition is the exit of health workers from the workforce permanently (leaving the health sector). Involuntary attrition occurs when a health worker leaves permanently due to a condition that would prevent her from continuing to work in the public sector workforce (e.g. serious illness, dismissal, death, or retirement). | Physicians: 9.8%  Clinical officers: 4.5%  Registered nurses: 5.3% Enrolled nurse: 4.5%  Registered and enrolled midwives: 4.5% | Physicians: 3.1% Clinical officers: 1.4%  Registered nurses: 1.7% Enrolled nurses: 1.4%  Registered and enrolled midwives: 1.4% |
| Tabatabai P, et al./2013 | The internal migration between public and faith-based health providers: a cross-sectional, retrospective and multicentre study from southern Tanzania. | To explore health worker migration from a hospital level perspective. An emphasis is placed on quantification, direction patterns and motives of internal migration between public and FBO facilities in southern Tanzania. | Tanzania/  Sub-national | Low | Internal migration: internal staff movement of health professionals from one health facility to another (excludes deaths, retirements or prolonged training). | - | Total staff  Transfer from public hospitals: 4.3%  Transfer from facility based organisations (FBO): 30.1%  Nurses:  Transfer from public hospitals: 7.6%  Transfer from FBO: 60.0% |
| Van D, et al./2008 | Appropriate training and retention of community doctors in rural areas: a case study from Mali. | Findings of an evaluation of the training course promoted by NGO (Santé Sud) and the Malian Rural Doctors Association, which addressed the following research questions: What are unmet training needs for rural general practitioners in Mali; What were effects of the orientation course on trainees, and; Did the course affect retention, and if so, how? | Mali / Nationwide (rural) | Low | Retention of GPs after 5 years of ending training (not clearly defined). Losses include: transfer to a different health facility, move to a specialised training or to an NGO. | - | Physicians:  Total: 15% (over 5 years)  2003-07: 50.0%  2004-07 : 23.0%  2005-07: 14.0%  2006-07: 19.0% |
| Woltmann EM, et al./2008 | The role of staff turnover in the implementation of evidence-based practices in mental health care. | To examine the turnover rates of teams implementing psychosocial evidence-based practices in public-sector mental health settings. It also explored the relationship between turnover and implementation outcomes in an effort to understand whether practitioner perspectives on turnover are related to implementation outcomes. | USA/ Sub-national (fifty-two sites across eight states) | High | Turnover: the total number of people leaving the team during the 24-month implementation period divided by the total number of original team positions. | - | Staff working in mental health care:  81.0% (Average 2 years)  Migration/transfer: 29.0%  Resignation: 57.0%  Termination: 12.0% |
| Zaman C./2010 | ASISP: Annual country report. Pensions, health and long-term care in Romania | Country annual report | Romania/ Nationwide | Upper-middle | n/a | - | Physicians migration: 10.0% |
| Zijlstra Ed E, Broadhead RL./2007 | The College of Medicine (COM) in the Republic of Malawi: towards sustainable staff development. | To describe the professional development and geographical distribution of the COM graduates after the first 15 years. To describe projected staff requirements with special reference to inflow of Malawian senior staff and decreasing dependence on expatriates. Lastly, to explore measures that may be effective to retain the graduates in the country while at the same time assuring the highest possible level of training and career opportunities. | Malawi/ Nationwide | Low | Retention (not clearly defined). | - | Physicians migration: 40.0% (over 15 years) |

***Studies reporting only on retention rates or % of health workers retained in a certain period of time were not included.**
